# Comparison of estimation capabilities of response surface methodology (RSM) with artificial neural network (ANN) in lipase-catalyzed synthesis of palm-based wax ester

**DOI:** 10.1186/1472-6750-7-53

**Published:** 2007-08-30

**Authors:** Mahiran Basri, Raja Noor Zaliha Raja Abd Rahman, Afshin Ebrahimpour, Abu Bakar Salleh, Erin Ryantin Gunawan, Mohd Basyaruddin Abd Rahman

**Affiliations:** 1 Faculty of Science, Universiti Putra Malaysia, 43400 UPM Serdang, Selangor, Malaysia; 2 Faculty of Biotechnology and Biomolecular Sciences, Universiti Putra Malaysia, 43400 UPM Serdang, Selangor, Malaysia

## Abstract

**Background:**

Wax esters are important ingredients in cosmetics, pharmaceuticals, lubricants and other chemical industries due to their excellent wetting property. Since the naturally occurring wax esters are expensive and scarce, these esters can be produced by enzymatic alcoholysis of vegetable oils. In an enzymatic reaction, study on modeling and optimization of the reaction system to increase the efficiency of the process is very important. The classical method of optimization involves varying one parameter at a time that ignores the combined interactions between physicochemical parameters. RSM is one of the most popular techniques used for optimization of chemical and biochemical processes and ANNs are powerful and flexible tools that are well suited to modeling biochemical processes.

**Results:**

The coefficient of determination (R^2^) and absolute average deviation (AAD) values between the actual and estimated responses were determined as 1 and 0.002844 for ANN training set, 0.994122 and 1.289405 for ANN test set, and 0.999619 and 0.0256 for RSM training set respectively. The predicted optimum condition was: reaction time 7.38 h, temperature 53.9°C, amount of enzyme 0.149 g, and substrate molar ratio 1:3.41. The actual experimental percentage yield was 84.6% at optimum condition, which compared well to the maximum predicted value by ANN (83.9%) and RSM (85.4%). The order of effective parameters on wax ester percentage yield were; respectively, time with 33.69%, temperature with 30.68%, amount of enzyme with 18.78% and substrate molar ratio with 16.85%, whereas R^2 ^and AAD were determined as 0.99998696 and 1.377 for ANN, and 0.99991515 and 3.131 for RSM respectively.

**Conclusion:**

Though both models provided good quality predictions in this study, yet the ANN showed a clear superiority over RSM for both data fitting and estimation capabilities.

## Background

Wax esters are long chain esters that are derived from fatty acids and alcohols with chain lengths of 12 carbons or more. The compounds have many potential applications due to their excellent wetting behavior at interfaces [1] and a non-greasy feeling when applied on skin surfaces. Wax esters are important ingredients in cosmetic formulations (cleansers, conditioners and moisturizers), pharmaceuticals (as an anti foaming agent in the production of penicillin), lubricants, plasticizers and polishes and other chemical industries [2].

Natural waxes originate from animals, vegetables and minerals. Many of the important commercial waxes contain rather high percentages of saturated wax esters, such as beeswax. Other raw materials for saturated and unsaturated wax esters are sperm whale and jojoba oil [2]. Since the naturally occurring wax esters are expensive and limited in access, the need to synthesize the compound has grown. Wax esters have been synthesized via chemical [3] and enzymatic reactions [4]. Enzymatic synthesis uses lower temperatures than chemical synthesis [2].

Wax esters can be produced by alcoholysis of vegetable oils such as palm oil. Palm oil consists of triacylglycerides, which are a combination of glycerol and different fatty acids. Enzymatic synthesis of wax esters from rapeseed fatty acid methyl ester [2] and lipase-catalyzed alcoholysis of crambe and camelina oil [5] have been reported.

One of the most important stages in a biological process is modeling and optimization to increase the efficiency of the process [[6,7] and [8]]. The classical method of optimization involves varying one parameter at a time and keeping the other constant. This technique is not only time-consuming but also does not depict the complete effects of the parameters in the process and ignores the combined interactions between the physicochemical parameters. In contrast, response surface methodology (RSM) is an effective statistical technique for developing, improving, and optimizing of complex process [[6] and [9]]. RSM is a collection of statistical and mathematical techniques that can be used to define the relationships between the response and the independent variables [10]. RSM defines the effect of the independent variables, alone or in combination, in the processes. In addition to analyzing the effects of the independent variables, this experimental methodology also generates mathematical model.

Although RSM has so many advantages, and has successfully been applied to study and optimize the enzyme synthesis of flavor ester [[7] and [11]] and biodiesel (fatty acid alkyl ester) [12] and also optimizing enzyme production from microorganisms [[13-15] and [16]], it is hard to say that it is applicable to all optimization and modeling studies. Baş and Boyacı [6] reported that the second-order polynomial equation was not suitable in explaining the effects of pH and substrate concentration on the initial reaction rate of the enzymatic reaction. Similar observations were made on the data of some RSM articles [[17,18] and [19]].

The past decade has seen a host of data analysis tools based on biological phenomena developed into well-established modeling techniques, such as artificial intelligence and evolutionary computing. Artificial neural networks (ANNs) are now the most popular artificial learning tool in biotechnology, with applications ranging from pattern recognition in chromatographic spectra and expression profiles, to functional analyses of genomic and proteomic sequences [20]. An artificial neural network is an information processing paradigm that is inspired by the way biological nervous systems, such as the brain, process information. Indeed an artificial neural network is a massively interconnected network structure consisting of many simple processing elements capable of performing parallel computation for data processing. The fundamental processing element of artificial neural networks (the artificial neuron, unit or nodes) simulates the basic functions of biological neurons [[6] and [21]].

In the present investigation, RSM and ANN analysis of enzymatic synthesis of wax esters from palm oil and oleyl alcohol was carried out using a commercial immobilized lipase.

## Results and discussion

Experimental design along with the observed responses is shown in Table 1.

**Table 1:** Experimental design used in RSM and ANN studies by using four independent variables showing observed values of percentage yield of wax esters synthesis

**Temperature (°C)**	**Time (h)**	**Molar ratio (mmol)**	**Amount of enzyme (g)**	**Actual yield (%) mean ± SD**
40	2.5	2	0.1	30.2 ± 0.17
60	2.5	2	0.1	32.9 ± 0.52
60	7.5	2	0.1	75.5 ± 0.82
40	2.5	4	0.1	41.8 ± 0.46
40	7.5	4	0.1	75.2 ± 1.12
60	7.5	4	0.1	72.9 ± 0.57
60	2.5	2	0.2	55.6 ± 0.23
40	7.5	2	0.2	80.8 ± 0.37
60	7.5	2	0.2	78.7 ± 0.19
60	2.5	4	0.2	60.6 ± 0.41
40	7.5	4	0.2	72.4 ± 0.6
60	7.5	4	0.2	68.6 ± 0.36
30	5	3	0.15	48.5 ± 0.18
70	5	3	0.15	48 ± 0.3
50	0	3	0.15	20 ± 0.15
50	10	3	0.15	80 ± 0.77
50	5	1	0.15	70.2 ± 0.66
50	5	5	0.15	72.5 ± 0.85
50	5	3	0.05	70.1 ± 1.21
50	5	3	0.25	82 ± 0.49
*50*	*5*	*3*	*0.15*	*81.6 ± 0.28*
*50*	*5*	*3*	*0.15*	*82.0 ± 0.51*
*50*	*5*	*3*	*0.15*	*82.4 ± 0.43*
*50*	*5*	*3*	*0.15*	*80.0 ± 0.45*
*50*	*5*	*3*	*0.15*	*82.5 ± 0.78*
*50*	*5*	*3*	*0.15*	*82.7 ± 0.39*
**40**	**7.5**	**2**	**0.1**	75.2 ± 1.11
**60**	**2.5**	**4**	**0.1**	45.5 ± 0.3
**40**	**2.5**	**2**	**0.2**	46.2 ± 0.26
**40**	**2.5**	**4**	**0.2**	55.4 ± 1.44

ANN training set: normal and italic (center points) numbers

ANN testing set: bold numbers

### Response surface methodology

Fitting the data to various models (linear, two factorial, quadratic and cubic) and their subsequent ANOVA showed that reactions of palm oil and oleyl alcohol were most suitably described with quadratic polynomial model (equation 1):

**Yield (%) = -334 + 9.05A + 31.8B + 32.4C + 508D - 0.0858A^2^- 1.29B^2 ^- 2.80C^2 ^- 655D^2 ^- 0.0725AB - 0.0471AC + 0.545AD - 1.49BC - 32.9BD - 32.4CD**

where A is the temperature; B the time; C the molar ratio; and D the amount of enzyme.

The computed model *F*-value of 232 was higher than tabular value of *F*_0.01(14,15) _= 3.56, implying the model are significant at 1% confidence level. The model also showed statistically insignificant lack of fit, as was evident from the lower computed *F *value (3.95) than the tabular *F*_0.01(10,5) _value (10.1) at 1% level. On the other hand, the pure error was very low, indicating good reproducibility of the data obtained. With very small *P*-value (0.0001) from the analysis of ANOVA and a suitable coefficient of determination (R^2 ^= 0.995), the quadratic polynomial model was highly significant and sufficient to represent the actual relationship between the response (percentage yield) and the significant variables (Table 2). Zhou *et al*. reported that satisfactory quadratic response models were obtained for the incorporation of caproic acid into rapeseed oil [22]. Similar model was shown by Shieh *et al*. [9] and Chen *et al*. [23] who determined the optimization of lipase-catalyzed synthesis for biodiesel (soybean oil methyl ester) and kojic acid mono laurate, respectively.

**Table 2:** ANOVA for joint test

**Source**	**Sum of squares**	**Degree of freedom**	**Mean square**	***F*-value**	**Prob > *F***
Model	9506	14	679	232	< 0.0001^a^
Temperature (*A*)	5.86	1	5.86	2	0.177^b^
Time (*B*)	5180	1	5180	1770	< 0.0001^a^
Molar ratio (*C*)	19.6	1	19.6	6.7	0.0206^a^
Amount of enzyme (*D*)	359	1	359	123	< 0.0001^a^
*A*^2^	2020	1	2020	690	< 0.0001^a^
*B*^2^	1790	1	1790	611	< 0.0001^a^
*C*^2^	216	1	216	73.7	< 0.0001^a^
*D*^2^	73.5	1	73.5	25.2	0.000154^a^
*AB*	52.6	1	52.6	18	0.000712^a^
*AC*	3.55	1	3.55	1.22	0.288^b^
*AD*	1.19	1	1.19	0.406	0.533^b^
*BC*	221	1	221	75.6	< 0.0001^a^
*BD*	270	1	270	92.5	< 0.0001^a^
*CD*	42.1	1	42.1	14.4	0.0017^a^
Residual	43.8	15	2.92		
Lack of fit	38.9	10	3.89	3.95	0.0714^b^
Pure error	4.93	5	0.985		
Cor total	9550	29			

^a ^Significant at "Prob > *F*" less than 0.05

^b ^Insignificant at "Prob > *F*" more than 0.05

### Artificial neural network

#### Effect of architecture and topology on neural network performance

The selection of an optimal neural-network architecture and topology is of critical importance for a successful application. Several neural-network architecture and topologies (the number of hidden neurons, connection types, learning algorithms and transfer functions of input and hidden layers) were tested for the estimation and prediction of lipase-catalyzed synthesis of palm-based wax ester. Table 3 summarizes the top five ANN models.

**Table 3:** The effect of different neural network architecture and topologies on R^2 ^and AAD in the estimation of lipase-catalyzed synthesis of palm-based wax ester obtained in the training and testing of neural networks

**Name**	**Model**	**Learning Algorithm**	**Connection Type**	**Transfer Function Output**	**Transfer Function Hidden**	**Training Set R**^2^	**Training Set AAD (%)**	**Testing Set R**^2^	**Testing Set AAD (%)**
C11	4-15-1	IBP^a^	MFFF^b^	Linear	Tanh^d^	1	0.002844	0.994122	1.289405
C15	4-15-1	IBP	MNFF^c^	Linear	Tanh	1	0.014932	0.979308	3.157524
C9	4-12-1	IBP	MNFF	Linear	Tanh	1	0.000794	0.934519	4.538254
C16	4-10-1	IBP	MNFF	Linear	Tanh	1	0.014686	0.937174	5.3352
C10	4-10-1	IBP	MFFF	Linear	Tanh	1	0.002646	0.904918	6.323364

^a ^Incremental Back Propagation

^b ^Multilayer Full FeedForward

^c ^Multilayer Normal FeedForward

^d ^Hyperbolic Tangent Function

#### The effect of learning algorithm and transfer function

Training a neural network model essentially means selecting one model from the set of allowed models that minimizes the cost criterion. We have tested different learning algorithms for training neural network models. All accepted models (RMSE < 0.01, R = 1 and DC = 1) have shown that incremental back propagation (IBP) was the most suitable learning algorithm for prediction of lipase-catalyzed synthesis of palm-based wax ester (Table 3).

According to IBP learning algorithm, in training, a set of inputs is presented to a network of randomly preassigned weights. Each neuron in the hidden and output layers first calculates the weighted sum of its inputs and passes the result through a transfer function to produce an estimate as output that corresponds to the input data set. The result is compared to the corresponding desired values and the error is back-propagated through the network to adjust the connection weights according to the learning rule. This procedure is repeated iteratively, until the predetermined target RMSE is reached [24].

The type of transfer function employed affects the neural network's learning rate and is instrumental in its performance. In the present work, among all employed transfer functions for hidden and output layers, accepted models (RMSE < 0.01, R = 1 and DC = 1) were produced by linear function for output layer and hyperbolic tangent (Tanh) or Gaussian function for hidden layer that between them, the best models have been obtained by hyperbolic tangent (Tanh) function.

#### Optimal number of hidden neurons

**Figure 1:**
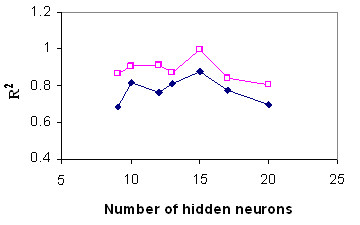
Optimal number of hidden neurons. Estimation of percentage yield of palm-based wax ester with neural networks of varying number of hidden neurons, tested with two example cases: incremental back propagation multilayer normal feedforward with Gaussian transfer function (blue diamond) and multilayer full feedforward incremental back propagation with Tanh transfer function (pink square).

Although it is important to select the optimal number of hidden neurons carefully, depending on the type and complexity of the task, this usually has to be done by trial and error. An increase in the number of hidden neurons up to a point usually results in a better learning performance. Too few hidden neurons limit the ability of the neural network to model the process, and too many may allow too much freedom for the weights to adjust and, thus, to result in learning the noise present in the database used in training [24]. We tested the effect of number of hidden neurons on the goodness of fit. The results of testing with the two sample experiments, evaluated statistically on the basis of the coefficient of determination (R^2^), are shown in Figure 1. In all examined cases, the optimum number of hidden neurons was 15, with an obvious increase in the calculation time and overfitting when too many hidden neurons were used. Then the 4-15-1 topology was chosen as the best topology for estimation of wax ester percentage yield.

#### Artificial neural network analysis of synthesis of palm-based wax ester

**Figure 2:**
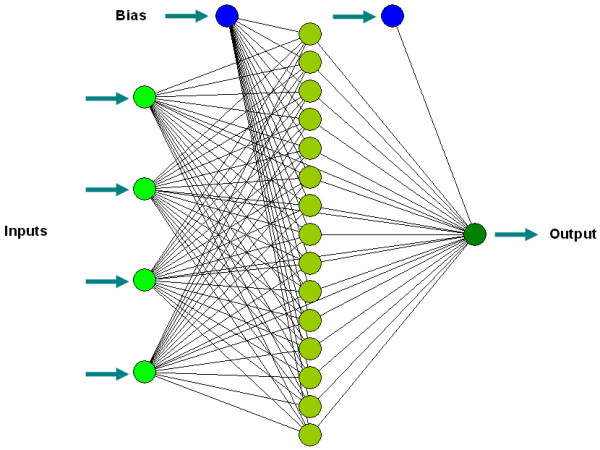
Neural network topology. Topology of multilayer full feedforward neural network for the estimation of lipase-catalyzed synthesis of palm-based wax ester.

The best ANN chosen in the present work was a multilayer full feedforward incremental back propagation network with Tanh transfer function (Table 3, C11) that consisted of a 4-15-1 topology (Figure 2). The optimized values of network for learning rate and momentum were 0.15 and 0.8, respectively. The learning was completed in RMSE = 0.00998, R = 1 and DC = 1. In the case of training data set, the coefficient of determination (R^2^) and absolute average deviation (AAD) were 1 and 0.002844, respectively, whereas for the testing data set, R^2 ^was 0.994122 and AAD was 1.289405 (Table 4) and for validating data sets R^2 ^and AAD were, 0.99998696 and 1.377, respectively (Table 5). Comparison of predicted and experimental values in training, testing and validating data sets, not only revealed capability of ANN in prediction of known data responses (the data that have been used for training) but also showed the ability of generalization for unknown data (the data that have not been used for training) and implying that empirical models derived from ANN can be used to adequately describe the relationship between the input factors and output in Lipozyme-catalyzed synthesis of wax ester from palm oil and oleyl alcohol.

**Table 4:** Predicted percentage yields by ANN and RSM models along with absolute deviation, R^2^and AAD

**ANN Predicted yield (%)**	**ANN Absolute deviation**	**RSM Predicted yield (%)**	**RSM Absolute deviation**
30.19981	6.36E-06	29.4	0.027211
32.89836	4.98E-05	34.4	0.043605
75.49839	2.13E-05	75.8	0.003958
41.79979	5.05E-06	42.8	0.023364
75.19847	2.04E-05	78	0.035897
72.89809	2.62E-05	72.5	0.005517
55.59765	4.22E-05	54.1	0.027726
80.79801	2.47E-05	80.3	0.006227
78.69785	2.74E-05	79.1	0.005057
60.59789	3.48E-05	59.2	0.023649
72.39832	2.33E-05	72.3	0.001383
68.59837	2.38E-05	69.3	0.010101
48.49916	1.73E-05	46.6	0.040773
47.99756	5.09E-05	48.6	0.012346
19.99912	4.42E-05	20.2	0.009901
79.99778	2.77E-05	81.9	0.023199
70.19804	2.79E-05	68.9	0.018868
72.49749	3.46E-05	72.5	0
70.09794	2.94E-05	67.6	0.036982
81.99761	2.92E-05	83.1	0.013237
*81.86748*	*3.08E-05*	*81.9*	*0.000366*
**74.07436**	**0.014969**	78.0	0.037234
**45.0392**	**0.010128**	45.9	0.00879
**46.27601**	**0.00165**	48.0	0.038961
**56.77555**	**0.02483**	55.0	0.00722

ANN training set R^2 ^= 1

ANN training set AAD (%) = 0.002844

RSM R^2 ^= 0.999619

RSM AAD (%) = 0.0256

**ANN testing set R**^2 ^= **0.994122**

ANN testing set AAD (%) = 1.289405

ANN training set: normal and italic (center points) numbers

ANN testing set: bold numbers

**Table 5:** Solution of optimum condition

**Expriment**	**Temperature (° and C)**	**Time (h)**	**Molar ratio (mmol)**	**Amount of enzyme (g)**	**ANN Predicted yield (%)**	**Actual yield (%)**	**RSM Predicted yield (%)**
1	53.9	7.38	3.41	0.149	83.9	84.6	85.4
2	54.6	7.33	2.07	0.123	83.3	81.9	85
3	55.6	6.69	3.09	0.181	84.5	85.2	84.9
4	46.8	5.72	3.57	0.195	81.1	80	83.9
5	50.2	5.31	2.66	0.155	80.7	79	83.5

ANN R^2 ^= 0.99998696

RSM R^2 ^= 0.99991515

ANN AAD (%) = 1.377

RSM AAD (%) = 3.131

#### Comparison of RSM and ANN predicted values

The predicted output values of RSM and ANN are shown in Table 4. Though both the models based on RSM and ANN preformed well and offered stable responses in predicting the combined interactions of the independent variables with respect to the response, yet the ANN based approach was better in fitting to the measured response in comparison to the RSM model.

#### Effect of parameters

**Figure 3:**
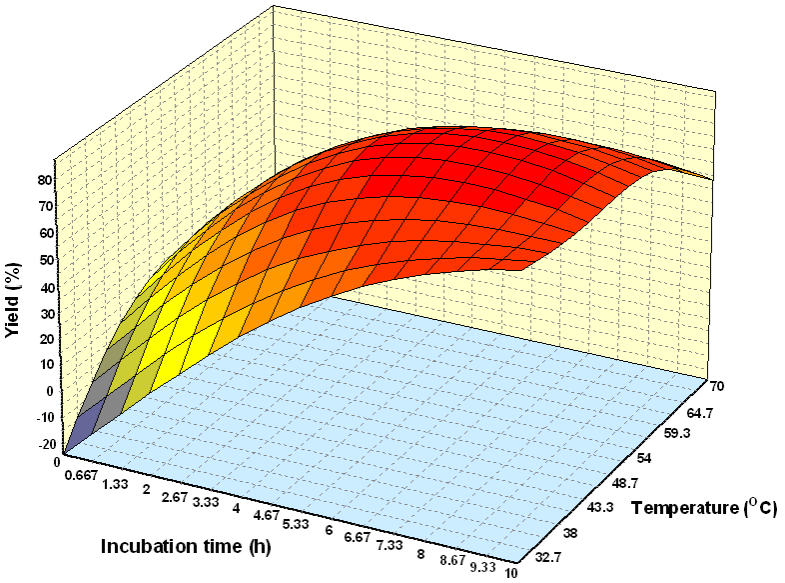
Three dimensional plot showing the effect of incubation time, temperature and their mutual effect on the synthesis of wax esters. Other variables are constant: enzyme, 0.15 g and molar ratio palm oil:oleyl alcohol, 1:3.

**Figure 4:**
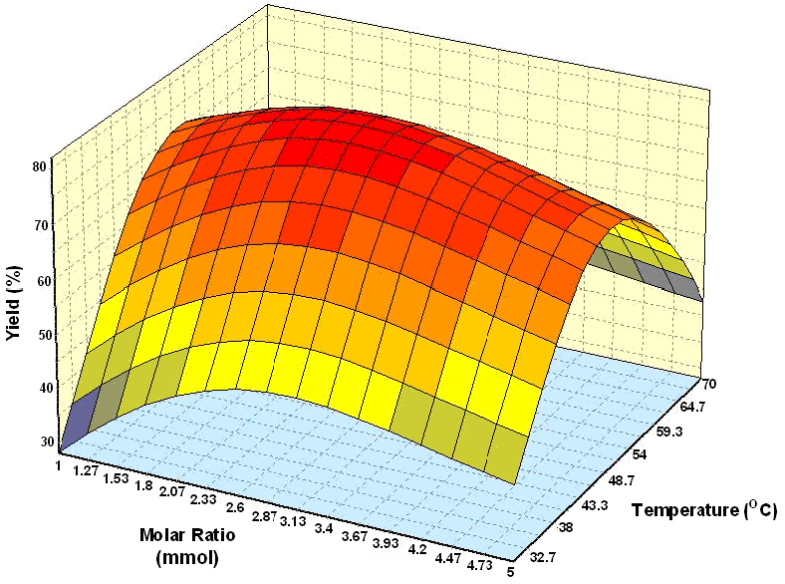
Three dimensional plot showing the effect of substrate molar ratio, temperature and their mutual effect on the synthesis of wax esters. Other variables are constant: enzyme, 0.15 g and incubation time, 5 h.

**Figure 5:**
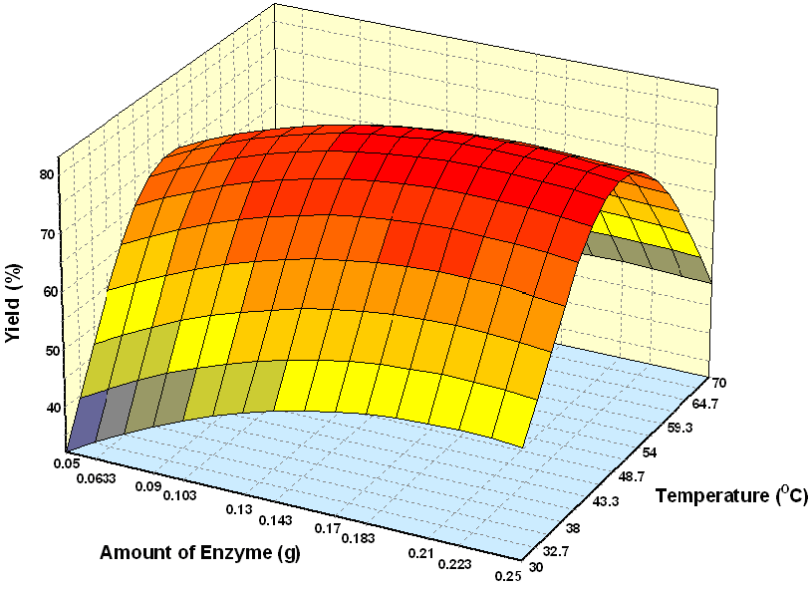
Three dimensional plot showing the effect of amount of enzyme, temperature and their mutual effect on the synthesis of wax esters. Other variables are constant: molar ratio palm oil:oleyl alcohol, 1:3 and incubation time, 5 h.

Figure 3 shows the three dimensional plots as function of time, temperature and interaction on wax ester synthesis at substrate molar ratio 1:3 and amount of enzyme of 1.50%. The percentage yield increased with an increase in incubation time. Reaction with temperature 50°C and time 7.5 h, led to the maximum percentage yield (over 80%). The percentage yield was increased from 40 to 50°C and decreased thereafter up to 60°C. The increase in percentage yield is an indication of the conformational change indicating greater unfolding of the enzyme at 50°C than at 30 and 60°C [25]. The effect of varying substrate molar ratio and reaction temperature on alcoholysis at constant reaction time (5 h), and amount of enzyme at 1.50% is as shown in Figure 4. Figure 5 represents the effect of varying amount of enzyme and reaction temperature on alcoholysis at 5 h and substrate molar ratio of 1:3. The typical plots are dome shaped. Many lipase-catalyzed esterification systems exhibit this type of plots [26]. In this type of plot, while in one axis there is a linear increase in alcoholysis, in the other axis there is increase only up to an extent, which decreases thereafter. This indicates that a critical temperature is involved up to which alcoholysis is favored and it is not so after that critical temperature. However percentage yield was lower at 55°C. Meanwhile, the low percentage yield at high substrate molar ratios (Figure 4) indicated that alcohols are terminal inhibitor of lipases and their effects could increase at high temperatures. Harikrisna *et al*. suggested that high temperature has reduced the operational stability of the enzyme [27]. Chiang *et al*. had reported an increase in temperature up to 55°C resulted in less alcoholysis at any given amount of enzyme because of the inactivation of enzyme at temperature over 55°C [11].

**Figure 6:**
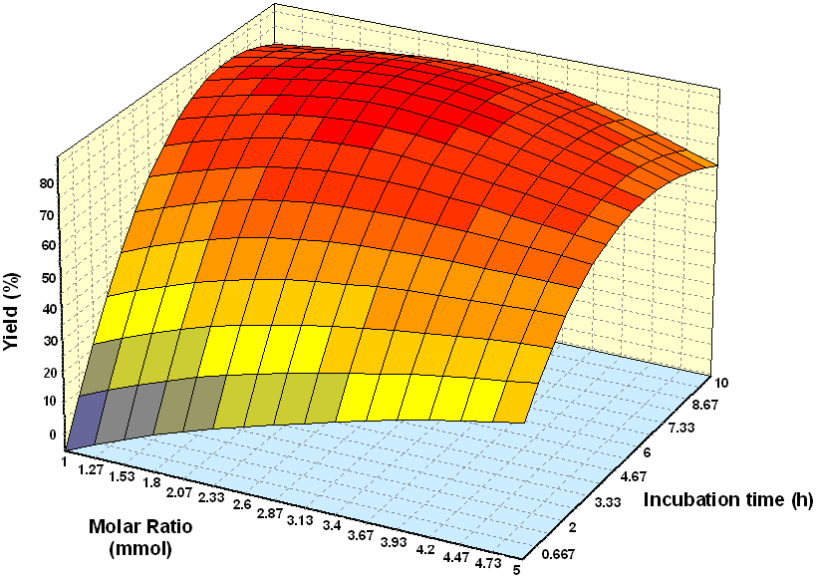
Three dimensional plot showing the effect of substrate molar ratio, incubation time and their mutual effect on the synthesis of wax esters. Other variables are constant: enzyme, 0.15 g and temperature, 50°C.

**Figure 7:**
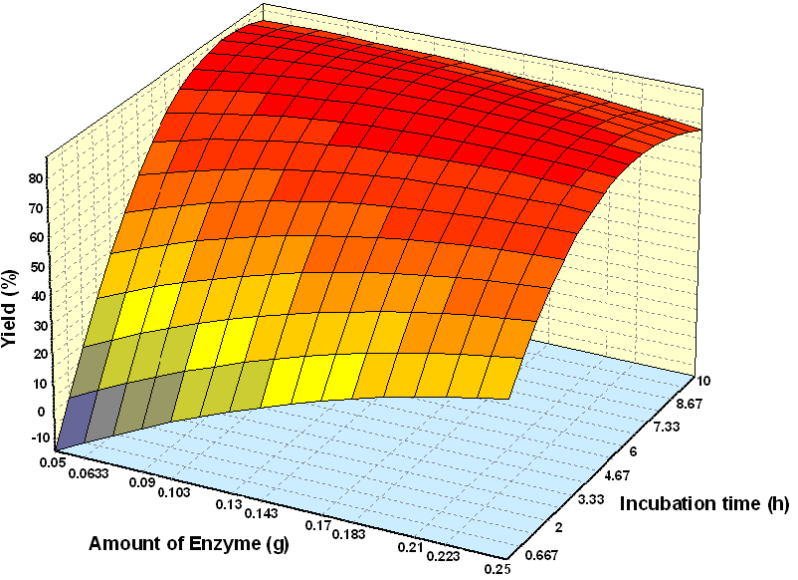
Three dimensional plot showing the effect of amount of enzyme, incubation time and their mutual effect on the synthesis of wax esters. Other variables are constant: molar ratio palm oil:oleyl alcohol, 1:3 and temperature, 50°C.

Figure 6 and Figure 7 depict the response surface plots as function of incubation time versus substrate molar ratio (palm oil:oleyl alcohol) and incubation time versus amount of enzyme, at temperature 55°C. A reaction with moderate substrate molar ratio 1:3 (palm oil:oleyl alcohol) and highest reaction time favored maximal yield and decreases up to substrate molar ratio 1:3.5. This may be due to at around critical molar ratio, the competing alcohol binding reduces the formation of the acyl-enzyme complex and thereby result in decrease in alcoholysis [26]. Kiran *et al*. reported that an enzymatic-catalyzed synthesis of lauroyl lactic acid had shown that the interaction of incubation time versus lactic acid concentration had a positive effect [28]. A linear increase in wax esters production with increase in amount of enzyme and incubation time was observed. The rate increased proportionally with enzyme loading. Similar trends for interaction of enzyme concentration and incubation time was reported by Hamsaveni *et al*. in their lipozyme-catalyzed esterification of isobutyric acid with isobutyl alcohol [7].

**Figure 8:**
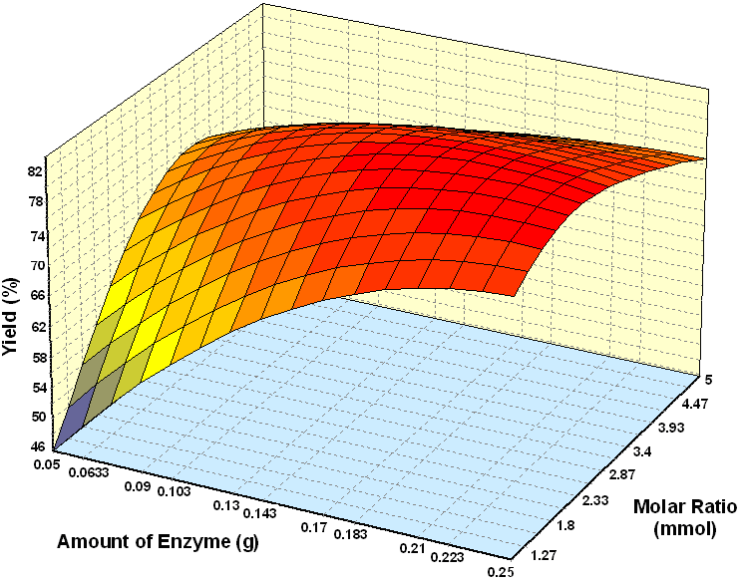
Three dimensional plot showing the effect of amount of enzyme, substrate molar ratio and their mutual effect on the synthesis of wax esters. Other variables are constant: incubation time, 5 h and temperature, 50°C.

Figure 8 represents the effect of varying amount of enzyme and substrate molar ratio on alcoholysis at 5 h and 50°C. At low amount of enzyme and low substrate molar ratio, the yield was lower. Reaction with high amount of enzyme and substrate molar ratio of 1:3 – 3.5 showed maximal percentage yields. The presence of higher amount of substrates generally increases the probability of substrate enzyme collision [29], and increasing amount of enzyme will lead to an increased percentage yield. This relationship holds when there are no limiting factors such as a low substrate concentration, presence of activators or inhibitors or mass transfer effect. The percentage yield was slightly decreased at substrate molar ratio 1:4. It is known that hydrophilic substrates have the capability of stripping off even the essential water from the enzyme surface, leading to insufficiently hydrated enzyme molecule and in turn to a decrease in enzyme activity [30]. However, two authors reported that even at high substrate levels and low enzyme concentration, high conversion could be achieved which is relevant from the economic point since the cost of enzyme is usually higher than that of substrate [[9] and [27]].

**Figure 9:**
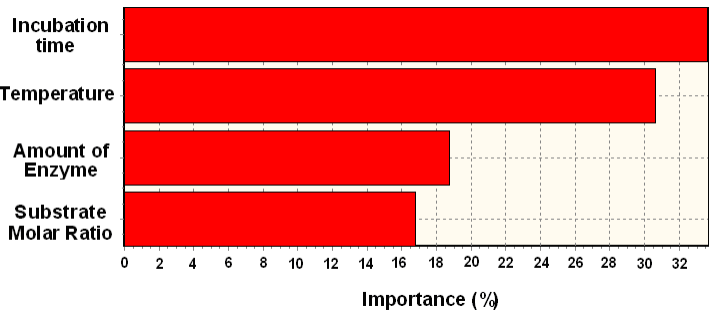
Importance of effective parameters on percentage yield of wax ester.

Finally, Figure 9 shows the importance of percentage of effective parameters on the percentage yield. Time with 33.69% is the most important factor on the percentage yield, temperature with 30.68%, amount of enzyme with 18.78% and substrate molar ratio with 16.85% are subsequent degrees of importance.

#### Optimization of reaction

The optimal conditions for the Lipozyme-catalyzed synthesis of wax esters were predicted as presented in Table 5 along with their predicted and actual values. Among the various optimum conditions, the highest percentage yield (85.2%) was from experiment 3. However, a reaction condition of 53.9°C, 7.38 h, substrate molar ratio 1:3.41 and 1.15% amount of enzyme (experiment 1) was chosen as the optimum condition, because experiment 3 used more enzymes to achieve highest percentage yield. Although experiment 1 used more substrates than experiment 3, enzyme is more expensive than substrate. If it was necessary to complete the synthesis within 7 h without the concern for cost, the time factor should be considered first, and then the other factors could be maximized. All the optimum conditions could be used to produce high percentage yield of wax esters. Attention to R^2 ^and AAD values between actual and estimated responses demonstrated a higher prediction accuracy of ANN compared to RSM.

The quantitative analysis of the products using GC showed that the alcoholysis of palm oil with oleyl alcohol produced esters with different chain length fatty acids. The composition of synthesized esters at optimum condition is presented in Table 6.

**Table 6:** Composition of ester produced from palm oil alcoholysis at optimum condition

Esters	Percentage (%)
Oleyl laurate	0.8 ± 0.13
Oleyl myristate	3.8 ± 0.60
Oleyl palmitate	35.5 ± 4.05
Oleyl stearate	4.5 ± 0.25
Oleyl oleate	33.3 ± 1.92
Oleyl linoleate	6.8 ± 0.39

**Total**	**84.6 ± 1.4**

## Conclusion

This study compared the performance of the RSM and ANN in the estimation of Lipozyme-catalyzed synthesis of wax ester from palm oil and oleyl alcohol. Though both models provided good quality predictions for the four independent variables (reaction time, temperature, amount of enzyme and substrate molar ratio) in terms of the percentage yield of wax esters, yet the ANN methodology showed a clear superiority over RSM as a modeling technique for data sets showing nonlinear relationships. As a modeling technique, artificial neural network was better than RSM for both data fitting and estimation capabilities. Regression-based response surface models require the order of the model to be stated (i.e., second, third or fourth order) [31], but unfortunately most of the packed program produced for the application of RSM use second order model equation and then the major drawback of RSM is to fit the data to a second order polynomial, while ANN tends to implicitly match the input vectors to the output vector [[31] and [32]]. Indeed ANN is a superior and more accurate modeling technique when compared to the RSM as it represents the nonlinearities in much better way [31].

On the other hand, neural networks also have the disadvantage of requiring large amounts of training data in comparison with RSM that offers a large amount of information from a small number of experiments. This advantage of RSM is because of its experimental design [10]. To overcome this ANN problem, in present study we used the RSM idea, and then a statistical experimental design, CCRD, was employed to reduce the number of experiments. Thus, ANN could be a very powerful and flexible tool for modeling the optimization process.

## Methods

### Materials

Immobilized lipase from *Mucor miehei *(Lipozyme IM) was produced by Novo Nordisk (Denmark). Palm oil (MW = 3 × average of saponification equivalent of palm oil) was obtained from Southern Edible Oil Sdn. Bhd. (Malaysia). Fatty acid compositions of Malaysian palm oil are 0.1 – 0.3% of lauric acid, 0.9 – 1.5% of myristic acid, 39.2 – 45.2% of palmitic acid, 3.7 – 5.1% of stearic acid, 37.5 – 44.1% of oleic acid and 8.7 – 12.5% of linoleic acid [33]. Oleyl alcohol was obtained from Fluka Chemika (Switzerland). Ester standards, oleyl laurate, oleyl myristate, oleyl palmitate, oleyl stearate, oleyl oleate, oleyl linoleate and methyl linoleate were obtained from Sigma Aldrich (USA). Hexane was obtained from J.T. Baker (USA). All other chemicals were of analytical grade.

### Experimental design

A five-level-four-factor central composite rotary design (CCRD) was employed in this study, requiring 30 experiments [34]. The fractional factorial design consisted of 16 factorial points, 8 axial points and 6 center points. The variable and their levels selected for the wax esters synthesis were: time (2.5 – 10 h); temperature (30 – 70°C); amount of enzyme (0.1 – 0.2 g) and substrate molar ratio (1 mmol palm oil to 1 – 5 mmol oleyl alcohol, 1:1 – 1:5). All experiments were carried out at the water activity equal to one.

The experimental data [35 points include CCRD design (Table 1) and optimization data (Table 5)] was divided into three sets: training set, testing set and validating set.

### Synthesis and analysis

Different molar ratios of palm oil and oleyl alcohol were added to 10 ml *n*-hexane, followed by different amounts of enzyme. The mixture of palm oil, oleyl alcohol and Lipozyme IM were incubated in a horizontal water bath shaker (150 rpm) at different reaction temperatures and reaction times. The reactions were analyzed by a gas chromatograph (Hitachi model G-3000, Tokyo, Japan), using an Rtx-65TG capillary column (30 m × 0.25 mm). Helium was used as the carrier gas at a flow rate 30 ml min-1. The temperature was programmed at 2 min at 150°C, 20°C min-1 to 300°C and 10 min at 300°C. The product composition was quantitated by an internal standard method with methyl linoleate as the internal standard. The concentrations of esters were calculated by equation 2:

***C*_*x *_= (*A*_*x*_/*A*_IS_)(*C*_IS_*D*_Rf IS_/*D*_Rf *x*_)**

where *C *is the amount of component *x *or internal standard, *A *is area for component *x *or internal standard and *D*_Rf _is detector response factor for component *x *or internal standard (*D*_Rfx _= *A*_*x*_/*C*_*x *_and *D*_Rf IS _= *A*_IS_/*C*_IS_).

The percentage yield of produced ester was calculated by equation 3:

**Percentage yield (%) = [ester produced (mmol)/palm oil used (mmol)] × 100**

### Response surface methodology analysis

The CCRD design experimental data was used for model fitting in RSM to find the best polynomial equation. This data was analyzed using design expert version 6.06 and then interpreted. Three main analytical steps: analysis of variance (ANOVA), a regression analysis and the plotting of response surface were performed to establish an optimum condition for the alcoholysis. Then, the predicted values obtained from RSM model, were compared with actual values for testing the model. Finally the experimental values of predicted optimal conditions (Table 5) were used as validating set and were compared with predicted values.

### Artificial neural network analysis

A commercial ANN software, NeuralPower version 2.5 (CPC-X Software) was used throughout the study. Multilayer normal feedforward and multilayer full feedforward neural networks were used to predict the percentage yields of palm-based wax ester that were trained by different learning algorithms (incremental back propagation, IBP; batch back propagation, BBP; quickprob, QP; genetic algorithm, GA; and Levenberg-Marquardt algorithm, LM). The network architecture consisted of an input layer with four neurons, an output layer with one neuron, and a hidden layer. Molar ratio of palm oil and oleyl alcohol, amount of enzyme, reaction temperature and reaction time were used as networks inputs and the percentage yield of palm-based wax ester, as target output. To determine the optimal network topology, only one hidden layer was used and the number of neurons in this layer and the transfer functions of hidden and output layers (sigmoid, hyperbolic tangent function, Gaussian, linear, threshold linear and bipolar linear) were iteratively determined by developing several networks. Each network was trained until the network root of mean square error (RMSE), average correlation coefficient (R) and average determination coefficient (DC) were lower than 0.01, equal to 1 and 1, respectively. Other parameters for network were chosen as the default values of the used software. At the start of the training, weights were initialized with random values and adjusted through a training process in order to minimize network error.

The CCRD design experimental data was divided into training and testing sets. For training, 26 points were used (Tables 1 and 4). One strategy for finding the best model is to summarize the data, it is well established [32] that in ANN modeling, the replicates at center point do not improve the prediction capability of the network because of the similar inputs. That is why we improved our model by using mean of center points instead of 6 center points (Tables 1 and 4, italic numbers). For testing the network, 4 remaining points were used (Tables 1 and 4, bold numbers). On the other hand, experimental values of predicted optimal conditions (Table 5) were used as validating set.

### Verification of estimated data

The estimation capabilities of the techniques, RSM and ANNs were tested. For this purpose, the estimated responses obtained from RSM and ANNs were compared with the observed responses. The coefficient of determination (R^2^) and absolute average deviation (AAD) were determined and these values were used together to compare ANNs to each other for finding the best ANN model, and the best ANN model with RSM. The AAD and R^2 ^are calculated by equations 4 and 5, respectively.

AAD={[∑i=1p(|yi,exp⁡−yi,cal|/yi,exp⁡)]/p}×100
 MathType@MTEF@5@5@+=feaafiart1ev1aaatCvAUfKttLearuWrP9MDH5MBPbIqV92AaeXatLxBI9gBaebbnrfifHhDYfgasaacH8akY=wiFfYdH8Gipec8Eeeu0xXdbba9frFj0=OqFfea0dXdd9vqai=hGuQ8kuc9pgc9s8qqaq=dirpe0xb9q8qiLsFr0=vr0=vr0dc8meaabaqaciaacaGaaeqabaqabeGadaaakeaacqqGbbqqcqqGbbqqcqqGebarcqGH9aqpcqGG7bWEcqGGBbWwdaaeWbqaaiabcIcaOiabcYha8jabbMha5naaBaaaleaacqqGPbqAcqGGSaalcyGGLbqzcqGG4baEcqGGWbaCaeqaaOGaeyOeI0IaeeyEaK3aaSbaaSqaaiabbMgaPjabcYcaSiabbogaJjabbggaHjabbYgaSbqabaGccqGG8baFcqGGVaWlcqqG5bqEdaWgaaWcbaGaeeyAaKMaeiilaWIagiyzauMaeiiEaGNaeiiCaahabeaakiabcMcaPiabc2faDjabc+caViabbchaWjabc2ha9jabgEna0kabigdaXiabicdaWiabicdaWaWcbaGaeeyAaKMaeyypa0JaeGymaedabaGaeeiCaahaniabggHiLdaaaa@6315@

where *y*_*i*,*exp *_and *y*_*i*,*cal *_are the experimental and calculated responses, respectively, and *p *is the number of the experimental run.

R2=1−Σi=1−n(model predictioni−experimental valuei)2Σi=1−n(average experimental value−experimental valuei)2
 MathType@MTEF@5@5@+=feaafiart1ev1aaatCvAUfKttLearuWrP9MDH5MBPbIqV92AaeXatLxBI9gBaebbnrfifHhDYfgasaacH8akY=wiFfYdH8Gipec8Eeeu0xXdbba9frFj0=OqFfea0dXdd9vqai=hGuQ8kuc9pgc9s8qqaq=dirpe0xb9q8qiLsFr0=vr0=vr0dc8meaabaqaciaacaGaaeqabaqabeGadaaakeaacqqGsbGudaahaaWcbeqaaiabikdaYaaakiabg2da9iabigdaXiabgkHiTmaalaaabaWaaSaaaeaacqqHJoWuaeaacqqGPbqAcqGH9aqpcqaIXaqmcqGHsislcqqGUbGBaaGaeiikaGIaeeyBa0Maee4Ba8MaeeizaqMaeeyzauMaeeiBaWMaeeiiaaIaeeiCaaNaeeOCaiNaeeyzauMaeeizaqMaeeyAaKMaee4yamMaeeiDaqNaeeyAaKMaee4Ba8MaeeOBa42aaSbaaSqaaiabbMgaPbqabaGccqGHsislcqqGLbqzcqqG4baEcqqGWbaCcqqGLbqzcqqGYbGCcqqGPbqAcqqGTbqBcqqGLbqzcqqGUbGBcqqG0baDcqqGHbqycqqGSbaBcqqGGaaicqqG2bGDcqqGHbqycqqGSbaBcqqG1bqDcqqGLbqzdaWgaaWcbaGaeeyAaKgabeaakiabcMcaPmaaCaaaleqabaGaeGOmaidaaaGcbaWaaSaaaeaacqqHJoWuaeaacqqGPbqAcqGH9aqpcqaIXaqmcqGHsislcqqGUbGBaaGaeiikaGIaeeyyaeMaeeODayNaeeyzauMaeeOCaiNaeeyyaeMaee4zaCMaeeyzauMaeeiiaaIaeeyzauMaeeiEaGNaeeiCaaNaeeyzauMaeeOCaiNaeeyAaKMaeeyBa0MaeeyzauMaeeOBa4MaeeiDaqNaeeyyaeMaeeiBaWMaeeiiaaIaeeODayNaeeyyaeMaeeiBaWMaeeyDauNaeeyzauMaeyOeI0IaeeyzauMaeeiEaGNaeeiCaaNaeeyzauMaeeOCaiNaeeyAaKMaeeyBa0MaeeyzauMaeeOBa4MaeeiDaqNaeeyyaeMaeeiBaWMaeeiiaaIaeeODayNaeeyyaeMaeeiBaWMaeeyDauNaeeyzau2aaSbaaSqaaiabbMgaPbqabaGccqGGPaqkdaahaaWcbeqaaiabikdaYaaaaaaaaa@B42A@

where *n *is the number of experimental data.

R^2 ^is a measure of the amount of the reduction in the variability of response obtained by using the repressor variables in the model. Because R^2 ^alone is not a measure of the model's accuracy, it is necessary to use absolute average deviation (AAD) analysis, which is a direct method for describing the deviations. Evaluation of R^2 ^and AAD values together would be better to check the accuracy of the model. R^2 ^must be close to 1.0 and the AAD between the predicted and observed data must be as small as possible. The acceptable values of R^2 ^and AAD values mean that the model equation defines the true behavior of the system and it can be used for interpolation in the experimental domain [32].

#### Optimization of reaction

The predicted optimal conditions could be easily calculated using model equation. The stationary point (minimum or maximum point) of a second order equation is the point where the first derivative of the function equals to zero:

Lety=f(x1,x2)and=β0+β1x1+β2x2+β11x12+β22x22+β12x1x2
 MathType@MTEF@5@5@+=feaafiart1ev1aaatCvAUfKttLearuWrP9MDH5MBPbIqV92AaeXatLxBI9gBamXvP5wqSXMqHnxAJn0BKvguHDwzZbqegyvzYrwyUfgarqqtubsr4rNCHbGeaGqiA8vkIkVAFgIELiFeLkFeLk=iY=Hhbbf9v8qqaqFr0xc9pk0xbba9q8WqFfeaY=biLkVcLq=JHqVepeea0=as0db9vqpepesP0xe9Fve9Fve9GapdbaqaaeGacaGaaiaabeqaamqadiabaaGcbaqbaeaabiqaaaqaauaabeqabmaaaeaaimqacaWFmbGaa8xzaiaa=rhaaeaacaWF5bacceGae4xpa0dcdmGaa0Nzaiaa=HcacaWF4bWaaSbaaSqaaiaa=fdaaeqaaGqabOGaeWhlaWIaa8hEamaaBaaaleaacaWFYaaabeaakiaa=LcaaeaacaWFHbGaa8NBaiaa=rgaaaaabaGae4xpa0Jae4NSdi2aaSbaaSqaaiaa=bdaaeqaaOGae43kaSIae4NSdi2aaSbaaSqaaiaa=fdaaeqaaOGaa8hEamaaBaaaleaacaWFXaaabeaakiab+TcaRiab+j7aInaaBaaaleaacaWFYaaabeaakiaa=HhadaWgaaWcbaGaa8NmaaqabaGccqGFRaWkcqGFYoGydaWgaaWcbaGaa8xmaiaa=fdaaeqaaOGaa8hEamaaDaaaleaacaWFXaaabaGaa8hiaiaa=jdaaaGccqGFRaWkcqGFYoGydaWgaaWcbaGaa8Nmaiaa=jdaaeqaaOGaa8hEamaaDaaaleaacaWFYaaabaGaa8hiaiaa=jdaaaGccqGFRaWkcqGFYoGydaWgaaWcbaGaa8xmaiaa=jdaaeqaaOGaa8hEamaaBaaaleaacaWFXaaabeaakiaa=HhadaWgaaWcbaGaa8Nmaaqabaaaaaaa@6FB4@

The stationary point is found by computing *∂y*/*∂x*_1 _and *∂y*/∂*x*_2 _and setting zero:

∂y/∂x1=β1+2β11x1+β12x2=0∂y/∂x2=β2+2β22x2+β12x1=0
 MathType@MTEF@5@5@+=feaafiart1ev1aaatCvAUfKttLearuWrP9MDH5MBPbIqV92AaeXatLxBI9gBaebbnrfifHhDYfgasaacH8akY=wiFfYdH8Gipec8Eeeu0xXdbba9frFj0=OqFfea0dXdd9vqai=hGuQ8kuc9pgc9s8qqaq=dirpe0xb9q8qiLsFr0=vr0=vr0dc8meaabaqaciaacaGaaeqabaqabeGadaaakeaafaqaaeGabaaabaacceGae8NaIylcbmGae4xEaKhcbeGae03la8Iae8NaIyRae4hEaG3aaSbaaSqaaiab9fdaXaqabaGccqWF9aqpcqWFYoGydaWgaaWcbaGae0xmaedabeaakiab=TcaRiab9jdaYiab=j7aInaaBaaaleaacqqFXaqmcqqFXaqmaeqaaOGae0hEaG3aaSbaaSqaaiab9fdaXaqabaGccqWFRaWkcqWFYoGydaWgaaWcbaGae0xmaeJae0Nmaidabeaakiab9Hha4naaBaaaleaacqqFYaGmaeqaaOGae8xpa0Jae0hmaadabaGae8NaIyRae4xEaKNae03la8Iae8NaIyRae4hEaG3aaSbaaSqaaiab9jdaYaqabaGccqWF9aqpcqWFYoGydaWgaaWcbaGae0Nmaidabeaakiab=TcaRiab9jdaYiab=j7aInaaBaaaleaacqqFYaGmcqqFYaGmaeqaaOGae0hEaG3aaSbaaSqaaiab9jdaYaqabaGccqWFRaWkcqWFYoGydaWgaaWcbaGae0xmaeJae0Nmaidabeaakiab9Hha4naaBaaaleaacqqFXaqmaeqaaOGae8xpa0Jae0hmaadaaaaa@6543@

The system of equations is solved to find the values of *x*_1 _and *x*_2_. To determine whether the stationary phase is minimum or maximum, the second derivative of the equation is used. If it is a negative value, the optimum point is a maximum but if it is a positive value, the optimum point is a minimum [32].

## Authors' contributions

MB, RNZRAR, ABS and MBAR conceived the idea of the study and experimental design. ERG performed most of the experiments described in this paper and contributed to RSM design and analysis. AE conceived the ANN design and analysis, compared the estimation capabilities of the RSM with ANN and drafted the manuscript. All authors read and approved the final manuscript.
